# A Case of Cognitive Decline Resulting from Aging, Temporal Lobe Epilepsy, and Environmental Factors

**DOI:** 10.1155/2019/9385031

**Published:** 2019-12-10

**Authors:** Nikhila Veluri

**Affiliations:** American University of Integrative Sciences, Department of Neuropsychiatry, Detwiller Pavilion at University of British Columbia, Vancouver, British Columbia, Canada

## Abstract

Cognitive functioning is imperative in our daily lives. It allows us to understand, process, and react appropriately to different situations. Aging has been linked to cognitive decline. The degree and rate of cognitive decline are crucial as they differentiate normal aging from dementia or memory loss secondary to medical conditions. A 63-year-old Caucasian woman with a 50-year history of temporal lobe epilepsy experienced memory difficulties in recent years. She was admitted voluntarily to the neuropsychiatry ward for a 3-day ambulatory electroencephalogram (EEG), which reported mild bitemporal structural or functional abnormality. The patient reported subjective seizure experiences that were not reflective of seizure activity on the EEG. Possible causes included panic attacks or other anxiety experiences. Routine laboratory test and magnetic resonance imaging results were unremarkable. During her hospital stay she showed improvement in cognitive functioning. However, anxiety continued to negatively impact her memory. We hypothesized that the memory impairments could have resulted from age, psychological factors, the patient's own expectations, pressure from the environment and history of TLE. We diagnosed the patient with mild cognitive impairment and adjustment disorder with anxiety. She was discharged with seizure and anxiety medication. This report highlights the importance of both age-related and disease-related variables when diagnosing patients with cognitive decline.

## 1. Introduction

Cognitive functioning is the process that allows humans to acquire and retain information, and hence create knowledge. There are many means and mechanisms through which knowledge can be developed. Cognitive function involves attention, memory, emotion, and executive functioning. Different regions of the brain are relied on more heavily for different cognitive functions. Human experiences are complex and our ability to understand, process, and react appropriately to situations largely depends on cognition. Therefore, cognition is critical in our daily activities.

When a person with high cognitive functioning suffers an imbalance of any of the cognitive areas, they may experience significant distress. Cognitive decline can be caused by a variety of conditions including, but not limited to, dementia, delirium, Alzheimer's disease (AD), mild cognitive impairment (MCI), temporal lobe epilepsy, and neurodegenerative diseases. We present a 63-year-old Caucasian woman with a history of temporal lobe epilepsy for the past 50 years who began experiencing memory difficulties in the past few years.

## 2. Case Presentation

A 63-year-old female was admitted to the neuropsychiatric ward for ambulatory electroencephalography (EEG) to be assessed for temporal lobe epilepsy. Two months prior to admission, she had undergone another EEG that did not show electrographic seizures. The patient reported that she had been experiencing seizures since the age of 10 years. She described the seizures as a déjàvu and dissociative feeling that was accompanied by olfactory hallucinations and smelling metal. In addition, she reported prodromal symptoms such as nausea, sweating, palpitations, blank stares, greying of vision, and feeling distant. The patient also reported a postseizure state of confusion that lasted for about 1 minute. She denied experiencing any lip-smacking or incontinence. Although the patient had been experiencing seizures her entire life, she had only begun seeking treatment approximately 5 years prior. She was taking valproic acid but had been recently switched to levetiracetam due to the side effects of valproic acid. Other than seizures, the patient denied any significant past medical history. The patient reported being sexually assaulted at the age of 7 and again in her late twenties. However, the exact ages at which these events occurred are uncertain since the patient was inconsistent with her recollection.

Her mother is alive and healthy in her late 80's without any cognitive issues. Her father passed away at the age of 92 due to a medical condition that was not otherwise specified. There is a family history of essential tremor on her maternal side.

The patient had worked in a large firm for over 10 years in which she had become a partner as a bankruptcy trustee. She had been asked to resign from her position two years prior due to unstable memory since she began forgetting scheduled meetings and conversations she had had with coworkers and clients. The patient had completed an undergraduate degree in commerce along with a certification for becoming a trustee. She has been married to her husband for over 30 years with no children. She drinks alcohol only occasionally and does not smoke or chew tobacco. She denies any recreational drug use (i.e., marijuana, cocaine, etc.).

The loss of her job, as well as her memory impairment, had caused her significant anxiety and low self-esteem. She reported being unable to remember to take her medications at the scheduled time or forgetting that she had taken them, causing her to take double the dosage. She also reported forgetting the names of people she recently met or forgetting having met them. Further, she reported confusion while navigating her surroundings and that she has previously gotten lost in familiar surroundings prompting her to call her husband for help. She also reported misplacing items around the house and difficulty finding things in the house despite having lived in the same house for many years. The patient reported that she could be cued to recall previous events; however, it was not always successful. These memory issues affected her confidence in socializing since she felt that she was being judged.

### 2.1. Mental Status Examination

The presented patient was a 63-year old, well-nourished, Caucasian female. She exhibited no signs of acute distress; however, she appeared slightly anxious. She was average-statured and was of slim built. She was well-groomed and wore casual clothing that was appropriate for the interview. Throughout the interview, she exhibited no difficulty in maintaining appropriate eye contact. She was alert and oriented to person and place; however, she exhibited having difficulty orienting to time; specifically, the month and date, which was consistent throughout her admission.

She did not exhibit any abnormal movements. Her mood was described as “good” and her affect was euphoric with full range. Her speech was normal in pressure, rate, rhythm, and volume.

The patient's thought processes were circumstantial and often repetitive. She also exhibited slight thought disorganization. Her thought content did not reveal any first-rank symptoms of schizophrenia or expansive cognitions; however, it was significant for ongoing low mood and anxiousness pertaining to her memory impairment. She believed that her memory was extremely poor in both verbal and nonverbal aspects. She expressed self-doubt and compared herself to her coworkers who were of the same age and were not having any difficulties with their jobs. She appeared to hold these beliefs with great intensity and rigidity.

Regarding her nonpsychotic preoccupations, she did not exhibit any suicidal or homicidal ideations, intentions, or plans. There was no evidence of perceptual abnormalities such as auditory or visual hallucinations or any other responses to internal stimuli during the interview. She was very insightful as evidenced by her recognition of her cognitive impairments (largely related to memory) and the fact that she sought proper diagnosis and treatment. Her judgment appeared to be good in that she did not demonstrate unsafe behaviors per se and she also proved to be cooperative and engaging.

To assess her cognition, the Montreal Cognitive Assessment (MoCA)—version 7.2 was conducted prior to admission. She scored 25/30, losing 5 points because of delayed recall. She had difficulties with visuospatial tasks; however, she was able to complete them with cueing. In 2013, she has previously performed the MOCA tests and had scored 29/30, losing a point for visuospatial tasks. In 2014, she scored 28/30 and in 2017, she scored 25/30, again losing 5 points for delayed recall. A day before her discharge, she completed another MOCA test and scored 25/30, losing 5 points for delayed recall. At this final test, she again displayed difficulties with visuospatial tasks but was able to accurately complete them with self-cueing.

### 2.2. Investigations

The patient completed an ambulatory 3-day EEG and the results were suggestive of mild underlying bitemporal structural or functional abnormality. The results showed an improvement from those of previous EEG she had undergone since there were no intermittent 5-6 Hz sharp and notched theta activity. The change to a levetiracetam regimen appeared to have led to an interim resolution of those changes.

She underwent routine blood tests (i.e., complete blood count with differential, liver function test, and tests to determine the levels of thyroid-stimulating hormone, electrolytes, albumin, B12, blood urea nitrogen, and creatinine) that showed results within the normal range. A magnetic resonance imaging (MRI) assessment performed in 2018 revealed minimal microvascular ischemic changes with no evidence of acute infractions or intracranial hemorrhages. The MRI findings did not indicate specific features that were the cause of the symptoms.

### 2.3. Neuropsychological Assessment

The patient had neuropsychological assessment was completed a few months prior to her hospital admission. Patient demonstrated good effort during the assessment. However, her test anxiety was high throughout the evaluation. The Wechsler Adult Intelligence Scale—Fourth Edition (WAIS-IV) was used for evaluation. Her overall intellectual functioning was uninterpretable due to a 27-point difference between her verbal (87^th^ percentile) and visual spatial index (27^th^ percentile). Her processing speed was lowest at 6^th^ percentile. Language related aspects of cognitive domain, such as task of verbal reasoning, common sense judgment, verbal reasoning between words, naming tasks, tasks providing definition of words and tasks of pronouncing words were scored at 91^st^ percentile or above by the patient. She demonstrated a perfect performance on a task of concept formation and mental flexibility. However, when she was asked an unfamiliar task with visuospatial loading, her performance was below average. Her lowest scores were on tasks of delayed recall (verbal or visual information). She also performed poorly on recognition testing on visual memory, tasks of block design and quick scanning for visual detail.

The assessment notes concluded stating, that the cognitive impairment present in the patient, has a neurological component present. However, her high degree of anxiety as well as low mood was periodically controlling her behavior. Patient was noted to be easily overwhelmed and become fatigued due to her high anxiety. This, led the patient to have impaired functioning with simple tasks, causing the patient to lose her confidence.

## 3. Discussion

Aging has been associated with a decline in cognitive functioning. However, the normality of the decline is dependent on its degree and onset. For example, the presence of acute memory impairment along with apraxia, aphasia, agnosia, or executive functioning impairment with attention deficit and fluctuation of the symptoms indicates delirium as the most likely diagnosis. However, if memory impairment presents without an acute onset, attention deficit, or fluctuations, then dementia is the most likely diagnosis [1]. Therefore, obtaining a detailed history of a patient's cognitive decline is imperative for proper diagnosis.

We diagnosed our patient as most likely having MCI. MCI is a vague diagnosis since it refers to the stage between normal cognitive functioning and the more serious decline of dementia or AD. MCI can be referred to as a decline of cognitive function from a previously normal functioning level. However, it is challenging to define the precise borders of the mild decline [1]. Before diagnosing a patient with MCI, it is important to rule out other possible etiologies that can influence cognition. For example, psychiatric conditions such as generalized anxiety and major depression have cognitive components; therefore, patients suffering from these conditions may experience cognitive impairment [2]. Furthermore, it is important to assess for electrolytes, specifically hyponatremia and hypercalcemia; thyroid disorders; cobalamin deficiency; and alcohol dependence, which have been associated with cognitive impairment [3]. We diagnosed our patient with MCI only after excluding these alternative conditions. A cross-sectional study conducted by Petersen et al. [4], assessed the cognitive changes between individuals with MCI, AD, and a control group as well as cognitive changes in the progression of MCI to AD. They used the following criteria for MCI diagnosis: (1) memory complaints, (2) normal activities of daily living, (3) normal general cognitive functioning, (4) abnormal memory for age, and (5) not demented. They found that subjects with MCI performed poorly compared to the controls; however, their performance was superior to that of subjects with AD. Compared to the controls, all measures of memory (i.e., measures of learning and delayed recall of word lists, paragraphs, and nonverbal material) were significantly impaired in individuals with MCI; moreover, there were comparable to those of subjects with AD. However, there was no significant difference in general cognition (measured by the Wechsler Adult Intelligence Scale-Revised) between patients with MCI and the controls. These findings are consistent with our findings since our patient's cognitive impairment solely affected her measures of memory. Even though we ruled out most etiologies for our patients' cognitive decline, we could not exclude generalized anxiety or temporal lobe epilepsy. We discuss next temporal lobe epilepsy and its association with cognitive functioning.

Temporal lobe epilepsy (TLE) has been associated with cognitive impairment. Studies have established that recurrent seizures, particularly TLE can impair all areas of cognitive function, including memory, executive functioning, language, and other cognitive features [5]. The medial temporal lobe is used to consolidate and convert our working memory (WM) to long-term memory (LTM). An impairment in this process leads individuals to have difficulty storing and retrieving information. Three important factors have shown an association with the severity of impairment of the WM in patients with TLE: left temporal lobe epilepsy, early onset TLE or increased number of TLE attacks and sclerosis of the hippocampus [5]. Early onset or increased frequency of TLE attacks can cause increased damage to the medial temporal lobe leading to issues with memory. In addition, TLE involves cell loss and gliosis in the hippocampus and the surrounding medial temporal lobe, parahippocampus, and entorhinal cortex, all of which play a crucial role in the formation and storage of memory [6]. A subset of TLE is transient epileptic amnesia (TEA), where memory loss is the primary concern during the ictal and interictal period [5]. Patients with TEA commonly report an accelerated rate of memory loss, with an emphasis on autobiographical memory [5]. Milton et al. [7] propose that the autobiographical memory loss could result from recurrent clinical or subclinical activity spreading from the medial temporal lobe to the neocortical areas, changing the neuronal dynamics and thus, disrupting and negatively impacting individual's autobiographical memories. Another study by Haag et al. [8], investigated the memory of public events (PE) and public fact knowledge in patients with TLE and compared it with that in a control group comprised of patients with other epilepsy syndromes and healthy people. They found that patients with TLE showed inferior memory of PE with significantly more memory deficit for recent PE. Patients with TLE showed less memory of PE from the onset of the illness, indicating that their memory impairment could be attributed to unsuccessful consolidation [8]. These findings are consistent with ours since our patient demonstrated difficulty remembering her past personal and autobiographical events (e.g., trips and events) and also recent public fact knowledge (i.e., the president of the United States of America prior to Donald Trump). Notably, our patient only sought treatment for her epilepsy five years prior. This could have played a role in the severity of damage to the medial temporal lobe, leading to memory impairment. The patient was taking antiseizure medication prior and during the course of her hospital stay, likely causing a stability in her seizures. Therefore, TLE cannot be ruled out from being an etiology for her cognitive impairment, irrespective of the fact that the ambulatory EEG did not reveal significant findings. Although, her most recent MRI displayed only a slight asymmetry of the medial temporal lobe with preserved hippocampal volume, it should be noted that not all patients with TLE will display temporal lobe atrophy [9]. A better predictor of epileptic foci was demonstrated by the hippocampal T2 relaxation time. Furthermore, entorhinal lesions appear to play a role in memory impairments, specifically in TLE patients [9]. Chang et al. [10] tested serological biomarkers such serum levels of heat shock protein 70 (HSP70), S100ß protein (S100ßP), neuronal specific enolase (NSE), plasma nuclear and mitochondrial DNA levels, to predict cognitive performance in TLE patients. Their results revealed that poorer cognitive performance in TLE patients showed higher HSP70 and S100ßP levels and higher frequencies of seizures equaled higher levels of HSP70, NSE, and S100ßP [10]. They also highlighted that serum HSP70 level correlated positively with the duration of epilepsy and inversely with memory scores in late registration, early recall score and hippocampal volume [10]. Thus, Chang et al. [10] suggested serum HSP70 to be considered as a stress biomarker for TLE patients. Future patients presenting with TLE and cognitive impairment with regards to memory, should be evaluated for aspects such as hippocampal T2 relaxation time, entorhinal lesions, and biomarker serum HSP70. These test could improve our current understanding of TLE and cognitive impairment.

Storage and retrieval of memories are multifaceted. Trauma, anxiety, social interactions, and high expectations can attribute to memory impairment. Memories are occasionally repressed by traumatizing events such as physical or sexual abuse. Ogle et al. [11], compared childhood sexual abuse (CSA) and specificity in autobiographical memory between adults and adolescents. Adolescents with a history of CSA reported less specific autobiographical memories compared with those without. In contrast, there was no significant difference in the autobiographical memory specificity between adults with a history of CSA and those without [9]. Although adults with a history of CSA did not demonstrate a lack of specificity in autobiographical memory, Macmillian et al. [12], found that anxiety and depressive disorders were significantly higher in individuals with either physical or sexual childhood abuse. This indicates an interplay between childhood abuse, anxiety, and memory impairment. A longitudinal study reported that anxiety symptoms and disorders are associated with poor cognitive performance showing a greater decline over time [13]. In addition, the study demonstrated a bidirectional association between anxiety symptoms and cognitive domains of processing speed and attention [13]. This can be explained by the fact that a decrease in cognitive performance can cause worry and distress, and hence further decreasing cognitive functioning. Further, a decline in cognitive performance can affect social functioning; therefore, causing individuals to avoid social settings [13]. Our patient also reported such behavior where she began avoiding settings where she felt judged for her decreased cognitive performance such as interactions with her past coworkers or friends who made judgmental remarks. This indicates the need for psychoeducation on cognitive aging since educating older individuals on the normal decline of cognitive performance with aging can help decrease their apprehension of what they perceive as deterioration of their current functioning level. In addition, anxiety treatment should be provided to these individuals to further decrease anxiety and help with cognitive functioning [13]. Wilson et al. [14], explored how negative social interactions impacted cognitive functioning. The assessed negative social interactions included neglect or rejection, unwanted intrusion or advice, failure of others to provide help, and unsympathetic or insensitive behaviors by others. They found that negative social interactions were positively correlated with the incidence of MCI and cognitive decline [14]. Although the exact mechanism of this interaction is unclear, our findings and those of previous studies indicate that stress can cause physiological changes that can impact cognitive functioning. Future studies should examine this relationship at a closer level.

It should be noted, that this study has its limitations. An anxiety scale and a depression scale such as a Hamilton Depression Scale or Hamilton Anxiety Scale should have been administered to the patient. With these scales, an accurate rating could have been measured leading to certainty in our diagnosis. In addition, an auditory verbal learning test, logic memory tests or Clinical Dementia Rating ought to have been performed, supplementary to the MoCA. Results from the previously mentioned tests would have further validated our diagnosis. As with any case report, it is difficult to generalize our findings. Associations among aging, temporal lobe epilepsy, anxiety, and cognitive decline do not imply causation and therefore such associations cannot be generalized. Furthermore, this was a retrospective study and was hence subject to recall bias.

This report illustrates the need for further studies of cognitive decline with aging, including variables such as TLE, trauma, anxiety, and other secondary medical conditions. Furthermore, we have demonstrated the need to consider both age-related and disease-related variables when diagnosing patients with cognitive decline and providing them with psychoeducation.

## 4. Conclusion

A decline in cognitive functioning is unavoidable with age. However, the age at onset of decline, rate of decline, and type of cognitive impairment should be considered when determining whether the diagnosis is MCI, dementia, or memory impairment secondary to underlying medical conditions. Currently, there are no FDA-approved pharmacological treatments for MCI. However, identifying stressors causing anxiety that may attribute to MCI is important since the cognitive impairment could be improved with proper guidance and treatment of the anxiety and stressors. Future long-term studies should assess the pharmacological regimens used to treat AD to determine their effects on patients with MCI. Keeping the brain active with exercises such as crosswords and reading has always been advocated for memory improvement. Today, the availability and access to the Internet and programs such as Lumosity may also improve memory performances [15]. Further research must be conducted to ensure the effectiveness of such programs. Recommending these programs to patients whose MCI is likely due to anxiety and stress should be cautioned since poor performance in these programs may further increase their anxiety and stress. Concomitantly, we did not advise our patient to try these internet programs. There is also a great need for further research in cognitive impairments in patients with TLE. An MRI was performed on our patient prior to her hospital admission and therefore, we did not find the necessity to repeat this procedure. However, as we mentioned previously, hippocampal T2 relaxation time, entorhinal lesions, and biomarker serum HSP70 could have highlighted clearer results of TLE and cognitive functioning.

Our patient presented with a history of TLE and cognitive changes that were indicative of MCI. During her hospital stay, she demonstrated a high level of functioning that was significantly better than that at her home (i.e., taking her medications at the correct time, walks in novel environments without getting lost), which had an interfering overprotective environment and was associated with past traumatic experiences. EEG conducted during her hospital stay showed evidence of bitemporal slowing without any seizure activity. Further, the patient had subjective experiences of seizure episodes that were not reflective of the seizure activity on EEG, which could have been either due to panic attacks or other anxiety experiences. Despite her EEG findings, we cannot eliminate the possibility that the patient could have had TLE, which was now controlled. These findings support the hypothesis that her memory impairments could have resulted from TLE, psychological factors, the patient's own expectations and pressure from the environment. Thus, because the patient's diagnoses already encompass TLE, we diagnosed the patient with MCI and adjustment disorder with anxiety. During her stay at the hospital, she was provided medications for her seizures and anxiety (Levetiracetam 500 mg, twice a day and Escitalopram 20 mg, every morning, respectively). The patient also participated in daily cognitive behavioral therapy helping her with her anxiety, low mood, and confidence. Since she showed improvement during her hospital stay, with decreased anxiety, decreased low mood and increased confidence levels, she was discharged with seizure and anxiety medication. Further, we provided her and her family with psychoeducation on MCI and adjusting expectations.
